# Association Between Sleep Apnea and Valvular Heart Diseases

**DOI:** 10.3389/fmed.2021.667522

**Published:** 2021-08-09

**Authors:** Corrado Pelaia, Giuseppe Armentaro, Sofia Miceli, Maria Perticone, Alfredo Francesco Toscani, Valentino Condoleo, Martina Spinali, Velia Cassano, Raffaele Maio, Benedetto Caroleo, Nicola Lombardo, Franco Arturi, Francesco Perticone, Angela Sciacqua

**Affiliations:** ^1^Department of Health Sciences, University Magna Græcia of Catanzaro, Catanzaro, Italy; ^2^Department of Medical and Surgical Sciences; University Magna Græcia of Catanzaro, Catanzaro, Italy

**Keywords:** sleep apnea, valvular heart disease, central sleep apnea, obstructive sleep apnea, echocardiography

## Abstract

**Background:** Although sleep respiratory disorders are known as a relevant source of cardiovascular risk, there is a substantial lack of trials aimed to evaluate the eventual occurrence of associations between sleep apnea (SA) and valvular heart diseases (VHD).

**Methods:** We recruited 411 patients referring to our sleep disorder unit, among which 371 had SA. Ninety-three subjects with SA also suffered from VHD. Physical examination, echocardiography, nocturnal cardio-respiratory monitoring, and laboratory tests were performed in each patient. Patient subgroups were comparatively evaluated through cross-sectional analysis.

**Results:** A statistically significant increase in the prevalence of VHD was detected in relation to high apnea hypopnea index (AHI) values (*p* = 0.011). Obstructive sleep apnea occurrence was higher in SA patients without VHD (*p* < 0.0001). Conversely, central and mixed sleep apneas were more frequent among SA patients with VHD (*p* = 0.0003 and *p* = 0.002, respectively). We observed a direct correlation between AHI and BMI values (*p* < 0.0001), as well as between AHI and serum uric acid levels (*p* < 0.0001), high sensitivity C-reactive protein (*p* < 0.0001), and indexed left ventricular end-diastolic volume (*p* < 0.015), respectively. BMI and VHD resulted to be the main predictors of AHI values (*p* < 0.0001).

**Conclusions:** Our study suggests that a significant association can occur between SA and VHD. It is clinically relevant that when compared to SA patients without VHD, higher frequencies of central and mixed apneas were found in subjects with SA and VHD. Moreover, after elevated BMI, VHD represented the second predictor of AHI values.

## Introduction

Respiratory sleep disorders in adults represent an extensive and heterogeneous group of clinical conditions of varying severity which include snoring, increased upper airway resistance syndrome, obstructive sleep apnea (OSA), central sleep apnea (CSA), mixed sleep apnea (MSA), and obesity-hypoventilation syndrome (1). Although sleep apnea (SA) is still an underestimated disease in the world population, it has a high incidence in Western countries (2).

SA is defined as a respiratory dysfunction or alteration that occurs during sleep, characterized by repeated episodes of either reduction (hypopnea) or total cessation (apnea) of airflow through the airways. The estimated prevalence of this disease is around 5–15% of the general population, with a peak incidence between the sixth and seventh decade of life; it mainly affects males, and to a lesser extent females (3). Indeed, SA represents an important independent risk factor for cardiovascular diseases (CVD) such as arterial hypertension (AH), ischemic heart disease, heart failure (HF), diabetes mellitus (DM), and stroke (4). Moreover, in patients with valvular heart diseases (VHD) SA is a risk factor for clinical worsening (5). It has also been shown that the general prevalence of sleep breathing disorders, as well as the distribution of central and obstructive apneas in patients with aortic stenosis, are similar to those found in patients with HF (6). CSA has been reported in most cases of moderate and severe sleep disorders, while OSA prevails in mild forms. As the degree of severity expressed by apnea hypopnea index (AHI) worsens, the prevalence of CSA increases with respect to OSA, regardless of left ventricular function (6).

SA can be associated with rheumatic and non-rheumatic VHD (7). In patients with HF and mitral regurgitation it has been observed that SA worsens the extent of regurgitating flow and increases the cavity size of the two atria, thereby predisposing to the onset of atrial fibrillation and to deterioration of systolic function (8). Moreover, CSA and less frequently OSA may worsen when are concomitant with moderate-to-severe VHD causing HF (6). With regard to OSA, apnea occurs as a result of upper airways collapse, due to a decreased intrathoracic pressure which is associated with an impaired ability of oropharynx dilator muscles to maintain pharynx patency. In subjects with SA and VHD, Cheyne-Stokes periodic breathing can decrease the tone of pharyngeal dilator muscles, with a consequent further reduction of upper airway lumen (9). Both CSA and OSA can contribute to deteriorate cardiovascular function, thereby participating in the overall worsening of patient's clinical conditions (10). Importantly, CSA is an additional pathogenic factor for progressive deterioration of cardiac impairment, correlated with significant changes in vascular stiffness and aortic distensibility, which are respectively increased and reduced in CSA patients (11). These parameters correlate with the early diagnosis of left ventricle anatomical damage, mainly due to an increase in myocardial mass (12). In SA-induced cardiovascular dysfunction, a key pathogenic role is also played by oxidative stress, responsible for an increased production of pro-inflammatory cytokines such as tumor necrosis factor alpha (TNF-α) and interleukin-8 (IL-8) (13). This pathophysiologic scenario favors endothelial damage and atherosclerosis, thus possibly worsening hemodynamic changes occurring in VHD (14, 15). It is thus very important to assess, in our study population, the potential contribution of AHI values to the overall cardiovascular risk related to SA, which likely represents a more reliable reference than sleep apnea syndrome (16).

On the basis of the above considerations, the aim of our present study has been to evaluate, in a large cohort of patients with breathing-related sleep disorders, the association between SA and VHD.

## Methods

### Study Design and Endpoints

The study population consisted of consecutive patients referring to the Sleep Disorder Unit of the Geriatrics Division, located at “Mater Domini” University Hospital of Catanzaro, Italy, from January 2018 to January 2020. We recruited 411 outpatients, including 303 men (73.7%) and 108 women (26.3%). None of them took drug therapy or substances that could interfere with sleep. Patients suffering from HF with either preserved or reduced ejection fraction, ischemic heart disease, atrial fibrillation, severe renal and hepatic failure, moderate to severe respiratory failure or anemia were excluded. All patients underwent evaluation through the Epworth sleepiness scale, a questionnaire which identifies the probability of falling asleep under certain circumstances, based on the following score: 1–6 (normal sleepiness), 7–8 (mean sleepiness), and 9–24 (abnormal sleepiness) (17). Complete physical examination, including assessments of body mass index (BMI), body surface area (BSA), and waist circumference (WC) were performed in each patient. Moreover, echocardiography and laboratory tests aimed to evaluate metabolic disorders were also carried out.

All subjects underwent nocturnal cardio-respiratory monitoring (CRM) (Somtè, Compumedics, Australia), equipped with five cables utilized to record electrocardiogram through two bipolar leads, a nasal cannula used to detect the flow-meter trace, a microphone which records snoring, two piezoelectric belts measuring thoraco-abdominal movements, a digital pulse oximeter assessing peripheral arterial oxyhemoglobin saturation (SpO_2_), and a gravity sensor able to localize patient's position. Heart rate (HR) values were also measured. All the recordings were examined by the same operator, and each event was judged to be either obstructive, central and/or mixed, apnoic and/or hypopnoic, according to the criteria of the American Academy of Sleep Medicine (18). In particular, hypopneas were defined on the basis of a scoring criterium which considered the threshold of 3% oxygen desaturation (18).

Sleep apneas were classified as either obstructive or central, on the basis of the presence or absence of respiratory effort, respectively (18). MSA was diagnosed when an initial absence of respiratory effort was followed by the resumption of respiratory effort (18). The eventual presence of SA was checked, and patient population was stratified on the basis of AHI values (Group 0: AHI <5, normal; Group 1: 5 ≤ AHI <15, mild SA; Group 2: 15 ≤ AHI <30, moderate SA; Group 3: AHI ≥ 30, severe SA) (18). SpO_2_ levels during sleep were evaluated by means of oxygen desaturation index (ODI), corresponding to the number of episodes of desaturation >3% per hour of sleep. Furthermore, we also measured time below 90% (TC90), corresponding to the percentage time of saturation below 90% (19).

Measurements of arterial blood pressure (BP) were performed with an aneroid sphygmomanometer at level of the left arm in supine position, after 5 min of quiet rest. Three or more BP evaluations were performed on three different occasions, separated by at least 2 weeks. Systolic and diastolic BP values were registered at the first appearance (phase I) and disappearance (phase V) of Korotkoff sounds, respectively. Baseline BP values were obtained from the mean of the last two of three consecutive recordings, performed at intervals of 3 min. Pulse pressure (PP) values were also calculated. Patients with systolic BP (SBP) >140 mmHg and/or diastolic BP (DBP) >90 mmHg, as well as those with normal BP values under treatment with antihypertensive agents, were considered to be hypertensive according to current guidelines (20).

All laboratory measurements were carried out after at least 12 h of fasting. Glycemia was determined by the glucose oxidase method (glucose analyzer, BeckmanCoulter, Milan). Diabetes was diagnosed using the oral glucose tolerance test (OGTT), based on administration of a 75 g glucose load, followed by measurement of 2-h post-load blood glucose levels. Blood levels of total cholesterol, low-density lipoprotein (LDL) cholesterol, high-density lipoprotein (HDL) cholesterol, and triglycerides were measured by enzymatic methods (Roche Diagnostics GmbH, Mannheim, Germany). Serum insulin levels were determined in duplicate by chemiluminescence tests (Roche Diagnostics GmbH, Mannheim, Germany). Insulin resistance was assessed using the homeostatic model assessment (HOMA), based on the concentrations of fasting glucose and insulin, according to the following formula: blood glucose (mmol/L) × insulin (mU/mL)/22.5. The aforementioned index was used because of its good correlation with the euglycemic clamp, which represents the gold standard of insulin sensitivity (21). Creatinine levels were measured using the Jaffe method. The estimation of glomerular filtration rate (eGFR) was performed using the new CKD-EPI (Chronic Kidney Disease Epidemiology Collaboration) equation, that is more accurate especially in subjects with preserved renal function (22). Serum uric acid (UA) levels were measured using URICASE/POD method (Boehringer Mannheim, Mannheim, Germany). The high sensitivity C-reactive protein (hs-CRP) was measured by the immunoturbidimetric method automated system (Cardio Phase hs-CRP, Milan, Italy).

All subjects underwent standard left ventricular ultrasonography in both M-mode (motion mode) and B-mode (two-dimensional mode), according to the recommendations of the American Society of Echocardiography (23). Recordings were made with the patient in left lateral decubitus, using a VIVID 7 Pro ultrasound system (GE Technologies, Milwaukee, Wisconsin, USA) and a 2.5 MHz transducer.

Echocardiographic examinations were carried out by the same expert operator to minimize measurement errors. However, the operator was not aware of patient's clinical data, and the values represented the average of at least three measurements. According to our ultrasonographic methods, the intra-operative variability coefficient is 3.85% for posterior wall thickness (PWd), 3.70% for interventricular septal thickness (IVSd), 1.50% for left ventricle internal diameter at end diastole (LVEDD) and end systole (LVESD), and 5.10% for left ventricular mass (LVM), respectively. Devereux's formula was used to calculate LVM, which was subsequently indexed for BSA (LVMI) (24).

Left ventricular ejection fraction (LVEF) was calculated by the Simpson biplane method according to the following formula: LVEF = [left ventricular end-diastolic volume (LVEDV)-LV end-systolic volume (LVESV)]/LVEDV ×100 as mean of two measures in four and two apical chambers. Both volumes were subsequently indexed for BSA and expressed in mL/m^2^. In addition to EF, left atrial diameter (LAD) was measured using M-mode or two-dimensional echocardiography, from the posterior aortic wall to the posterior left atrial wall, in the parasternal long-axis view at the end-ventricular systole. Right ventricular systolic parameters were also assessed, by calculating the systolic pulmonary arterial pressure (S-PAP) estimate. The diameter and collapsibility of the inferior vena cava during the inhalation-expiratory phase, in subcostal projection, were used to estimate the right atrial pressure. Tricuspid regurgitant velocity (TRV) was assessed by continuous Doppler at level of the atrioventricular plane of the tricuspid valve, in projection with the four apical chambers or, in the case of eccentric jets, in parasternal short axis: therefore, S-PAP was derived through the Bernoulli equation. Diastolic dysfunction was evaluated by recording pulse-wave Doppler patterns at the mitral valve, in order to obtain early (E) and late (A) diastolic filling velocities from the 4-chamber view. The echocardiographic study of VHD and their grading was carried out according to guidelines, utilizing continuous doppler through a multi-parametric approach and apical window, using off axis approaches when appropriate (25). Only moderate and severe degrees of VHD were considered.

### Statistical Analysis

The differences in terms of anthropometric, clinical and biological parameters among the four different AHI groups, stratified with respect to their severity, were evaluated by one-way ANOVA for continuous variables followed by Bonferroni *post-hoc* test for multiple comparisons. Student's *t*-test for unpaired data was used to compare continuous variables between patients with or without VHD. Chi-square test was used for nominal data. Differences were considered to be significant for *p*-values < 0.05. A linear regression analysis was performed on the SA population, with the aim of investigating the eventual correlation between AHI values, (considered as a dependent variable) and different covariates. Subsequently, the variables significantly related to AHI were included in a stepwise multiple regression model. Statistical analysis was carried out using SPSS V20.0 program for Windows (SPSS Inc., Chicago, Illinois, USA).

## Results

In regard to the 411 enrolled patients, 40 subjects were characterized by normal AHI values, 93 suffered from mild SA, 116 had moderate SA, and 162 were found to have severe disease. Table 1 shows anthropometric, hemodynamic and biochemical parameters of the entire study population, stratified according to AHI values. There were no statistically significant differences in terms of age, SBP, DBP, PP, HR, blood glucose levels, total-, HDL- and LDL-cholesterol levels, as well as in terms of history of smoke, AH, diabetes, chronic obstructive pulmonary disease (COPD). For many of these parameters, the lack of differences can be mainly due to current pharmacological therapies. Conversely, patients with higher AHI values had greater BMI and WC, and lower mean SpO_2_ values (*p* < 0.0001). With regard to metabolic and biochemical parameters, high AHI values were associated with elevated fasting insulin levels (*p* < 0.0001), HOMA values (*p* < 0.027), serum UA concentrations (*p* < 0.0001), and hs-CRP levels (*p* < 0.0001). Furthermore, patients with severe SA had lower eGFR values (*p* < 0.001). Bonferroni's *post-hoc* analysis confirmed that patients who suffered from severe SA had worse BMI and WC, as well as worse renal function and metabolic profile. Table 2 highlights the percentage of patients with moderate to severe VHD within the whole study population, and with respect to AHI values. A statistically significant increase in the occurrence of VHD was detected in patients with high AHI values (*p* = 0.011). On the contrary, there was no statistically significant difference among groups with regard to the different VHD subtypes.

**Table 1:** Anthropometric, hemodynamic and biohumoral characteristics, stratified according to AHI values.

**Variables**	**All**	**Group 0**	**Group 1**	**Group 2**	**Group 3**	***P***
	**(*n* = 411)**	**(*n* = 40)**	**(*n* = 93)**	**(*n* = 116)**	**(*n* = 162)**	
Age, *years*	61.3 ± 10.7	61 ± 10.5	61.1 ± 9.9	61.6 ± 11.1	61.3 ±11	0.979
Gender, *m/f*	303/108	24/16	60/33	78/38	141/21	<0.0001*
BMI, *kg/m^2^*	33.9 ± 7.1	29.4 ± 5.6	33.1 ± 5.6	33.6 ± 6.8	35.7 ± 7.8	<0.0001^#^
WC, *cm*	111.9 ± 14.8	102.0 ± 13.3	110.7 ± 12.8	111.4 ± 15.4	115.7 ± 14.7	<0.0001^+^
Smokers, *n (%)*	96 (23.3)	14 (35)	25 (26.9)	26 (22.4)	31 (19.1)	0.148*
AH, *n (%)*	292 (71)	28 (70)	66 (70.9)	82 (70.7)	116 (71.6)	0.053*
Obesity, *n (%)*	281 (68.4)	17 (42.5)	63 (67.7)	78 (67.2)	123 (75.9)	0.0007*
Diabetes, *n (%)*	134 (32.6)	10 (25)	24 (25.8)	39 (33.6)	61 (37.6)	0.176*
COPD, *n (%)*	37 (9)	4 (10)	9 (9.7)	11 (9.5)	13 (8)	0.955*
SpO_2_, *(%)*	93.4 ± 1.5	94.4 ± 1.8	93.3 ± 1.3	93.3 ± 1.5	93.3 ± 1.5	<0.0001^x^
SBP, *mmHg*	131.2 ± 15.9	129.0 ± 16.5	131.0 ± 17.4	130.2 ± 12.4	132.5 ± 17.2	0.518
DBP, *mmHg*,	77.6 ± 9.9	76.3 ± 9.6	77.5 ± 10.3	76.6 ± 9.2	78.6 ± 10.1	0.364
PP, *mmHg*	53.6 ± 12.9	52.6 ± 13.0	53.5 ± 13.7	53.4 ± 10.8	53.9 ± 13.7	0.953
HR, *bpm*	70.7 ± 10.2	69.1 ± 9.9	69.8 ± 10.5	72.3 ± 10.2	70.4 ± 10.8	0.315
Glycemia, *mg/dL*	109.2 ± 34.6	99.2 ± 17.5	104.4 ± 20.7	111.8 ± 38.1	112.8 ± 40.9	0.067
Insulinemia, *mU/mL*	16.0 ± 8.3	13.9 ± 5.8	13.0 ± 6.6	16.4 ± 7.4	19.0 ± 9.8	<0.0001^‡^
HOMA	3.9 ± 1.9	3.1 ± 1	3.7 ± 1.6	4.3 ± 2.4	4.0 ± 2	<0.027^°^
Cholesterol, *mg/dL*	179.9 ± 39.9	193 ± 42.0	179.7 ± 41.7	178.3 ± 37.4	177.9 ± 39.3	0.184
HDL, *mg/dL*	46.9 ± 20.2	51.5 ± 14.6	47.4 ± 12.1	45.6 ± 11.8	46.4 ± 28.2	0.443
LDL, *mg/dL*	118.3 ± 38.3	126.4 ± 39.5	118.4 ± 41.0	115.5 ± 35.6	118.2 ± 38.4	0.500
eGFR, *mL/min/1.73m^2^*	88.7 ± 20.7	88.8 ± 18.2	95.2 ± 20.4	88.3 ± 15.8	84.7 ± 21.9	0.001^a^
Serum UA, *mg/dL*	5.8 ± 1.5	4.9 ± 0.8	5.4 ± 1.5	5.9 ± 1.5	6.1 ± 1.5	<0.0001^b^
hs-CRP, *mg/dL*	3.9 ± 3.3	2.5 ± 1.3	3.0 ± 2.2	3.9 ± 2.4	4.8 ± 3.8	<0.0001^c^

Data are expressed as mean ± SD; (*) chi-square test.

*AHI, apnea hypopnea index; BMI, body mass index; WC, waist circumference; AH, arterial hypertension; COPD, chronic obstructive pulmonary disease; SpO_2_, peripheral arterial oxyhemoglobin saturation; SBP, systolic blood pressure; DBP, diastolic blood pressure; PP, pulse pressure; HR, heart rate; HOMA, homeostatic model assessment; HDL, high-density lipoprotein; LDL, low-density lipoprotein; eGFR, estimated glomerular filtration rate; UA, uric acid; hs-CRP, high-sensitivity C-reactive protein*.

*Group 0, AHI <5; Group 1, 5 ≤ AHI < 15; Group 2, 15 ≤ AHI < 30; Group 3, AHI ≥ 30*.

*Bonferroni post-hoc analysis*:

# *Group 0 vs. 1, p = 0.024; Group 0 vs. 2, p = 0.006; Group 0 vs. 3, p < 0.0001*.

x *Group 0 vs. 1, p < 0.001; Group 0 vs. 2–3, p < 0.0001*.

° *Group 0 vs. 2, p = 0.044*.

+ *Group 0 vs. 1, p = 0.019; Group 0 vs. 2, p = 0.008; Group 0 vs. 3, p < 0.0001*.

‡ *Group 0 vs. 3. p = 0.014*.

a *Group 1 vs. 3, p < 0.0001*.

b *Group 0 vs. 2, p = 0.003; Group 0 vs. 3, p < 0.0001; Group 1 vs. 3, p = 0.002*.

c *Group 0 vs. 3, p < 0.0001; Group 1 vs. 3, p < 0.0001*.

**Table 2:** VHD prevalence, stratified according to AHI values.

**Variables**	**All**	**Group 0**	**Group 1**	**Group 2**	**Group 3**	***p***
	**(*n* = 411)**	**(*n* = 40)**	**(*n* = 93)**	**(*n* = 116)**	**(*n* = 162)**	
VHD*, n (%)*	98 (23.8)	5 (12.5)	16 (17.2)	24 (20.7)	53 (32.7)	0.011
Mitral insufficiency, *n*	26	2	6	8	10	0.359
Mitral stenosis, *n*	2	0	0	0	2	0.698
Aortic insufficiency, *n*	22	1	4	8	9	0.505
Aortic stenosis, *n*	14	0	0	1	4	0.828
Tricuspid insufficiency, *n*	5	0	0	1	4	0.827
Others, *n*	29	2	4	5	18	0.616

*VHD, valvular heart diseases; AHI, apnea hypopnea index*.

*Group 0, AHI < 5; Group 1, 5 ≤ AHI <15; Group 2, 15 ≤ AHI <30; Group 3, AHI ≥ 30*.

Subsequently, the analysis was conducted only in the SA population, including 93 patients with VHD and 278 without VHD, respectively. The two groups were compared for the different variables. This population sample approximately reflects the main rate of VHD patients, detected among all subjects referring to our center for sleep-related respiratory disorders during a 2-year period. In particular, within the group of 93 subjects with VHD, 15 (3.65% of overall population) had severe disease, and 78 (18.98% of overall population) suffered from moderate disease, respectively. Table 3 shows the anthropometric features, biochemical characteristics, and echocardiographic parameters of SA patients, with or without VHD. There were no statistically significant differences between the two groups in terms of BMI, WC, UA, PP, hs-CRP, and hemoglobin levels. Age, HOMA values, COPD and obesity frequency were higher in patients with VHD, while eGFR and SpO_2_ were lower. There were no differences between SA patients with or without VHD in regard to the occurrence of AH, smoking habit, and diabetes. Furthermore, also drug treatments were not significantly different. In particular, the intake of antihypertensive, antiplatelets, antidiabetic, and lipid lowering drugs was similar between the two groups. In regard to the overall study population, 123 (29.93%) patients were taking loop diuretics, 8 (1.95%) spironolactone, 287 (69.83%) renin–angiotensin–aldosterone system inhibitors, 205 (49.88%) calcium antagonists, and 82 (19.95%) beta-receptor blockers. No patient was taking sacubitril-valsartan, anticoagulants, cardiac glycosides, ivabradine, or other antiarrhythmic drugs. Because COPD was more frequent in SA patients with VHD, obviously inhaled drugs were significantly more used in this group (data not shown). When considering echocardiographic parameters, SA patients with VHD had higher values of LVEDD, LVESD, and LVEDV/BSA. Moreover, LVMI, LAD, S-PAP, EF, and E/A ratio were significantly higher in SA patients with VHD.

**Table 3:** Anthropometric, biohumoral and echocardiographic characteristics of SA patients, with or without VHD.

**Variables**	**All**	**VHD (+)**	**VHD (-)**	***p***
	**(*n* = 371)**	**(*n* = 93)**	**(*n* = 278)**	
Age, *years*	61 ± 11	66.3 ± 10.4	59.7 ± 10.3	0.0001
BMI, *kg/m^2^*	34.4 ± 7.1	35.5 ± 6.2	34.1 ± 7.3	0.166
WC, *cm*	113.0 ± 14.6	115.6 ± 14.0	112.1 ± 14.7	0.403
Smokers, *n (%)*	82 (22.1)	24 (25.8)	58 (20.9)	0.319*
Hypertension, *n (%)*	264 (71.2)	70 (75.3)	194 (69.8)	0.319*
Obesity, *n (%)*	264 (71.2)	75 (80.6)	189 (67.9)	0.029*
Diabetes, *n (%)*	124 (33.4)	29 (31.2)	95 (34.2)	0.594*
COPD, *n (%)*	37 (9)	19 (20.4)	18 (6.5)	0.0001*
PP, *mmHg*	53.7 ± 12.8	53.9 ± 14.7	54.1 ± 12.1	0.832
HOMA	3.9 ± 2	4.5 ± 2.8	3.7 ± 1.6	0.038
eGFR, *ml/min/1.73m^2^*	88.6 ± 20.2	84.7 ± 20.4	90.0 ± 20.0	0.040
Serum UA, *mg/dL*	5.8 ± 1.5	5.9 ± 1.7	5.9 ± 1.5	0.233
hs-CRP, *mg/dL*	4.1 ± 3.1	4.0 ± 3.0	4.1 ± 3.1	0.881
Hb, *mg/dL*	14.1 ± 4.5	13.4 ± 1.6	14.8 ± 7.4	0.571
LVMI, *g/m^2^*	115.9 ± 32.8	128.7 ± 40.3	111.7 ± 28.9	0.001
EF, %	60.8 ± 3.8	61.9 ± 3.3	60.4 ± 3.8	0.0001
E/A ratio	0.9 ± 0.3	1 ± 0.3	0.8 ± 0.2	0.0001
S-PAP, *mmHg*	32.3 ± 9.8	35.4 ± 11.5	31.2 ± 8.9	0.002
LVEDD, *cm*	5.3 ± 0.6	5.4 ± 0.6	5.2 ± 0.5	0.008
LVESD, *cm*	3.6 ± 0.6	3.8 ± 0.7	3.5 ± 0.5	0.003
LAD, *cm*	4.2 ± 0.6	4.6 ± 0.6	4.1 ± 0.6	0.0001
LVEDV/BSA, *mL/m^2^*	47.9 ± 21.7	54.2 ± 25.5	45.4 ± 19.6	0.031
SpO_2_, *(%)*	93 ± 2	92.1 ± 1.1	93.8 ± 1.4	0.0001

Data are expressed as mean ± SD; (*) chi-square test.

*SA, sleep apnea; VHD, valvular heart diseases BMI, body mass index; WC, waist circumference; COPD, chronic obstructive pulmonary disease; PP, pulse pressure; HOMA, homeostatic model assessment; eGFR, estimated glomerular filtration rate; UA, uric acid; hs-CRP, high-sensitivity C-reactive protein; Hb, hemoglobin; LVMI, left ventricular mass indexed; EF, ejection fraction; S-PAP, systolic pulmonary arterial pressure; LVEDD, left ventricle internal diameter at end diastole; LVESD, left ventricle internal diameter at end systole; LAD, left atrium diameter; LVEDV, left ventricular end-diastolic volume; BSA, body surface area; SpO_2_, peripheral arterial oxyhemoglobin saturation*.

**Figure 1:**
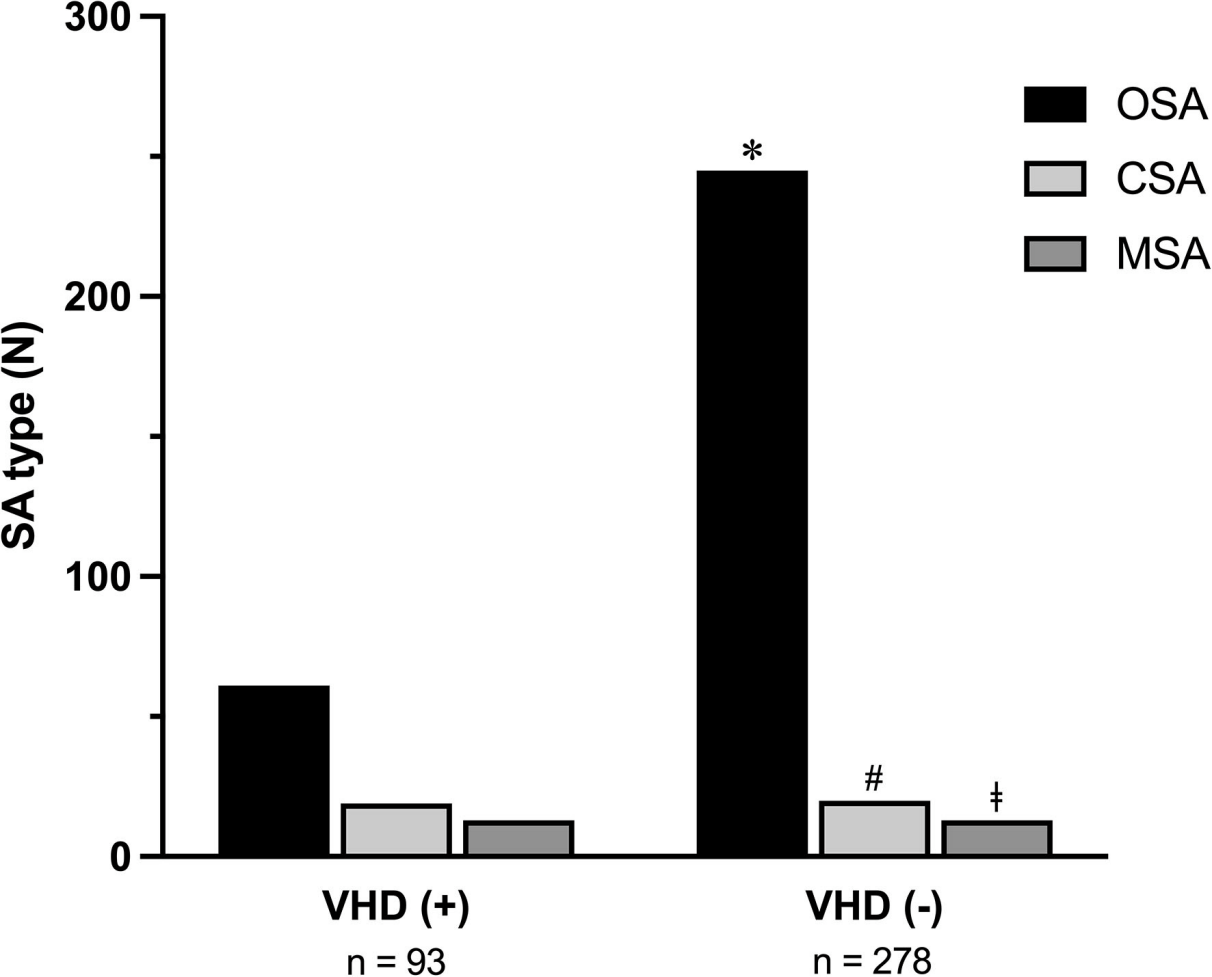
Occurrence of OSA, CSA, and MSA in SA patients with or without VHD. OSA, obstructive sleep apnea; CSA, central sleep apnea; MSA, mixed sleep apnea; SA, sleep apnea; VHD, valvular heart disease. *p < 0.0001; ^#^*p* < 0.0003; ^‡^*p* = 0.002.

In relation to the different types of apneas, it is noteworthy that the 93 SA patients with VHD included subjects with either OSA (61), or CSA (19), or MSA (13). The 278 SA patients without VHD included subjects with either OSA (245), or CSA (20), or MSA (13) (Figure 1). OSA occurrence was higher in SA patients without VHD (*p* < 0.0001). On the contrary, CSA and MSA were more frequent among SA patients with VHD (*p* = 0.0003 and *p* = 0.002, respectively) (Figure 1).

In regard to SA population, Table 4 shows the linear regression analysis of AHI values, considered as a dependent variable, related to different covariates such as age, BMI, PP, HOMA, eGFR, UA, hs-CRP, and LVEDV/BSA, respectively. We observed an inverse and statistically significant correlation between AHI and age (*r* = −0.091; *p* = 0.040), as well as between AHI and eGFR (*r* = −0.135; *p* = 0.005). We also found a direct and statistically significant correlation between AHI and BMI values (*r* = 0.326; *p* < 0.0001), as well as between AHI and serum UA levels (*r* = 0.200; *p* < 0.0001), hs-CRP (*r* = 0.237; *p* < 0.0001), and LVEDV/BSA (*r* = 0.112; *p* < 0.015) respectively. Conversely, no significant correlation was detected between AHI and either HOMA or PP (Table 4).

**Table 4:** Linear regression analysis focused on AHI as dependent variable in SA patients.

**Variables**	***R***	***p***
Age, *years*	−0.091	0.040
eGFR, *mL/min/1.73 m^2^*	−0.135	0.005
BMI, *kg/m^2^*	0.326	<0.0001
HOMA	0.039	0.228
Serum UA, *mg/dL*	0.200	<0.0001
hs-CRP, *mg/dL*	0.237	<0.0001
LVEDV/BSA, *mL/m^2^*	0.112	0.015
PP, *mmHg*	0.015	0.386

*AHI as dependent variable*.

*AHI, apnea hypopnea index; SA, sleep apnea; eGFR, estimated glomerular filtration rate; BMI, body mass index; HOMA, homeostatic model assessment; UA, uric acid; hs-CRP, high-sensitivity C-reactive protein; LVEDV, left ventricular end-diastolic volume; BSA, body surface area; PP, pulse pressure*.

Furthermore, study parameters which according to the linear regression analysis were found to be significantly associated with AHI as dichotomous variables, such as smoking habit, gender and VHD presence, were included in a stepwise multiple regression model to determine AHI independent predictors. BMI and VHD resulted to be the main predictors of AHI, thus accounting for 10.6 and 9.1% of its changes, respectively. This model also included hs-CRP, serum UA levels and eGFR, justifying 2.9, 1.1, and 2.8% of AHI variations, respectively. Overall, the entire model explains 28.1% of AHI changes (Table 5).

**Table 5:** Stepwise multiple regression analysis focused on AHI as dependent variable in SA patients.

**Variables**	**Partial *r*^**2**^ (%)**	**Total *r*^**2**^ (%)**	***p***
BMI, *kg/m^2^*	10.6	10.6	<0.0001
VHD, *yes/no*	9.1	19.7	<0.0001
hs-CRP, *mg/dL*	2.9	22.6	<0.0001
eGFR, *mL/min/1.73 m^2^*	2.8	28.1	0.001
Age, *years*	1.6	24.2	0.007
UA, *mg/dL*	1.1	25.3	0.026

*AHI as dependent variable*.

*AHI, apnea hypopnea index; SA, sleep apnea; BMI, body mass index; VHD, valvular heart disease; hs-CRP, high-sensitivity C-reactive protein; eGFR, estimated Glomerular Filtration Rate; UA, uric acid*.

## Discussion

The results of this single-center, observational study suggest that patients suffering from SA are characterized by a frequent association with VHD. In particular, higher is the AHI value, greater is the probability of detecting VHD as SA comorbidities. Moreover, according to our current investigation, VHD have emerged as one of the most important predictive factors of AHI entity, second only to BMI.

These findings are consistent with literature data, showing that patients with OSA and CSA are exposed to an increased cardiovascular risk (4). In fact, regardless of the different underlying pathophysiological mechanisms, causing recurrent episodes of hypoxemia, apnea contributes to the propagation of systemic inflammation, which is associated with hemodynamic changes, leading to an enhanced cardiovascular risk (26). In this regard, we herein show that high AHI values are associated with an increased occurrence of VHD. Indeed, AHI values lesser than 5 and higher than 30 were associated with VHD percentages of 12.5 and 32.7%, respectively. Moreover, in SA patients with VHD we recorded worse echocardiographic data such as increased LVMI, indexed LVEDV and S-PAP. According to several previous studies, the above changes can be closely correlated with the occurrence of VHD and the concomitant alterations of S-PAP and LVEDV/BSA (27, 28). Taken together, the above changes suggest that when SA and VHD coexist, several echocardiographic parameters worsen, especially those referring to increased measures of cardiac mass and atrial/ventricular diameters and volumes. These concepts have been already highlighted by previous studies, which however focused their attention on patients characterized by different features when compared to ours. Indeed, SA has been investigated in subjects with VHD associated with either HF (7) or rheumatic diseases (29), whereas we chose to search for VHD in patients with sleep respiratory disorders.

Our present results could be explained on the basis of common epidemiologic and pathogenic aspects shared by SA and VHD. Indeed, oxidative stress, inflammation, and endothelial dysfunction are main pathobiological determinants of SA (30, 31). Such changes cause inflammatory cell infiltration of upper airways, as well as vascular alterations which contribute to the association between SA and cardiovascular risk. Within this context, VHD acquire a prominent importance, given the key role played by oxidative stress, inflammation, and endothelial dysfunction also in the development and progression of cardiac valve impairment (32). In particular, the pro-inflammatory cytokines TNF-α, interleukin-6, and interleukin-17A are involved in the pathogenesis of both SA and VHD (33, 34). These considerations are probably true not only for rheumatic VHD, but also for non-rheumatic ones (34, 35). Because we herein show that the association frequency of SA and VHD increases in parallel with AHI increment, it can be reasonably argued that intensification of oxidative stress and inflammation favors the concomitance of upper airway obstruction and cardiac valve dysfunction. In addition to inflammatory changes, also the enhanced autonomic adrenergic tone can be implicated in the association of SA with VHD (36).

In our present study, OSA occurrence resulted to be higher than CSA or MSA in both SA patients with and without VHD. We also found that CSA and MSA variants were more frequent in subjects with VHD, when compared to those without VHD. Noticeably, CSA and VHD are characterized by relevant changes in vascular stiffness and aortic distensibility, which undoubtedly contribute to impair heart function (11).

After BMI, VHD was the second predictive factor of AHI variations. In this regard, it is possible to infer that distinct pathophysiological mechanisms underlie CSA and OSA, thus explaining the different frequencies of such apnea subtypes in patients with or without VHD. OSA is mostly related to the collapse of upper airways, due to a laxity of pharyngeal muscle tissue and to a concomitant increase of visceral adipose tissue (37). Anyway, statistical analysis was corrected with regard to BMI. In addition, BMI values did not result to be statistically different between SA patients with or without VHD. Therefore, OSA appears to be poorly correlated with hemodynamic changes caused by VHD. Conversely, CSA develops as a hemodynamic compensation mechanism in patients with moderate-to-severe VHD. In particular, in VHD patients nighttime supine position favors the redistribution of circulating blood volume, thus increasing pulmonary pressure in the capillary district; the consequent compensative hyperventilation results in stimulation of pulmonary vagal receptors (6). Hyperventilation also decreases arterial carbon dioxide partial pressure (PaCO_2_) below the apneic threshold (35 mmHg), thereby preventing the stimulation of brainstem breath centers, and causing CSA. Breath arrest increases PaCO_2_ and decreases arterial oxygen partial pressure (PaO_2_), thus leading to a compensatory resumption of breathing (9). However, this chain of events is not specific of VHD and cannot apply to all VHD patients. Serum UA levels were found to be a significant parameter in the multivariable regression model. Of course, a collinearity between serum UA variable and BMI variable cannot be ruled out.

It is very interesting to point out that all enrolled patients did not have HF. Hence, identification of such VHD patients among subjects with sleep related disorders is quite relevant, because an adequate treatment of SA could contribute to prevent the cardiovascular risk and the potential progressive evolution toward HF.

A limitation of our study regards the relatively small sample size of SA patients with VHD, which did not allow to detect relevant differences referring to clinical manifestations of breathing sleep disorders between stenotic valves and valve regurgitation, as well as to appreciate any difference related to VHD severity. Thus, the primary endpoint of a future extension of the present study could refer to a detailed analysis of such differences.

In conclusion, when compared to SA patients without VHD, higher numbers of CSA and MSA were found in subjects with SA and VHD enrolled in this observational investigation. Moreover, after elevated BMI, VHD represented the second predictor of AHI values. These findings reasonably suggest that VHD play a relevant role in the pathogenesis of SA. Therefore, our results may have a significant impact on clinical practice. Indeed, all physicians involved in the management of SA patients should be aware of the importance of a careful search for the eventual concomitant presence of VHD, especially but not only regarding people with high AHI. In fact, early detection of VHD as an important SA comorbidity might solicit an integrated therapeutic approach aimed to slow down the progressive combined deterioration of both respiratory and cardiac functions.

**Figure 2:**
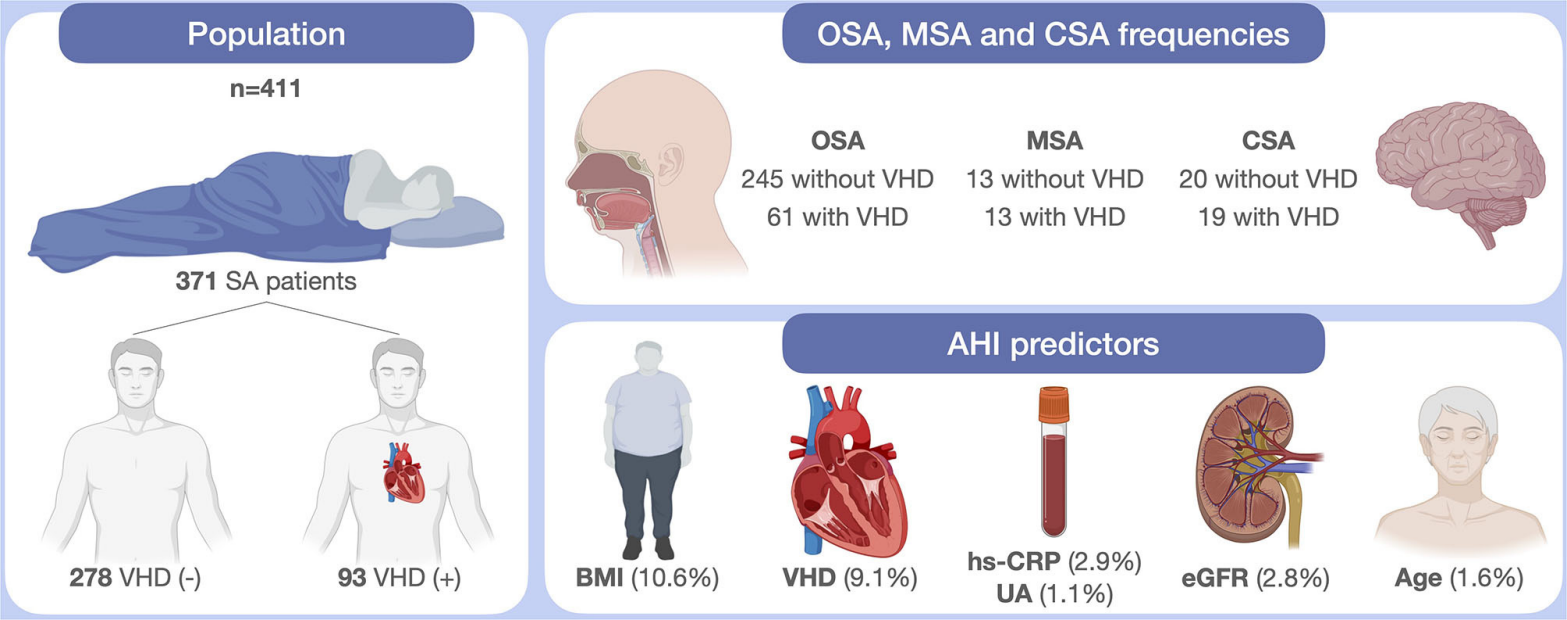
Schematic illustration of study findings. SA, sleep apnea; VHD, valvular heart disease; OSA, obstructive sleep apnea; MSA, mixed sleep apnea; CSA, central sleep apnea; AHI, apnea hypopnea index; BMI, body mass index; hs-CRP, high-sensitivity C-reactive protein; UA, uric acid; eGFR, estimated Glomerular Filtration Rate. This original figure was created by the authors using “BioRender.com.”

The main results of this study are schematically depicted in Figure 2.

## Data Availability Statement

The raw data supporting the conclusions of this article will be made available by the authors, without undue reservation.

## Ethics Statement

The studies involving human participants were reviewed and approved by Ethical Committee of Calabria Region, Italy (code protocol number 2012.63). The patients/participants provided their written informed consent to participate in this study.

## Author Contributions

All authors contributed to design and carry out the study protocol, as well as to write the text and draw the figures.

## Conflict of Interest

The authors declare that the research was conducted in the absence of any commercial or financial relationships that could be construed as a potential conflict of interest.

## Publisher's Note

All claims expressed in this article are solely those of the authors and do not necessarily represent those of their affiliated organizations, or those of the publisher, the editors and the reviewers. Any product that may be evaluated in this article, or claim that may be made by its manufacturer, is not guaranteed or endorsed by the publisher.
